# A multiplicity of identities: The intersections of cranial vault modification, paleodiet, and sex in San Pedro de Atacama, Chile

**DOI:** 10.1002/ajpa.24619

**Published:** 2022-09-15

**Authors:** Christina M. Torres, Kelly J. Knudson, William J. Pestle

**Affiliations:** ^1^ Department of Anthropology & Heritage Studies University of California Merced USA; ^2^ Instituto de Arqueología y Antropología Universidad Católica del Norte San Pedro de Atacama Chile; ^3^ Center for Bioarchaeological Research, School of Human Evolution and Social Change Arizona State University Tempe Arizona USA; ^4^ Department of Anthropology University of Miami Coral Gables Florida USA

**Keywords:** Andes, bioarchaeology, stable carbon and nitrogen isotope analyses, strontium isotope analysis

## Abstract

**Objectives:**

Intentional cranial modification and diet serve as markers of identity. Here, we explore the intersection between the body and social persona in the San Pedro oases through the complex relationship(s) between these markers and other aspects of society and the individual.

**Methods:**

Skeletal remains of 1190 individuals were analyzed for evidence of intentional head shaping and classed as unmodified, tabular, or annular. Stable carbon and nitrogen isotope analyses of bone (*n* = 203) focused on the contribution of C_3_ plants, C_4_/CAM plants, beans, and terrestrial animal meat per the Bayesian model, FRUITS. Strontium isotope data from dental enamel was considered for more detailed analyses.

**Results:**

Cranial modification was present in 520 individuals (43.7%; *N* = 1190). Modification was significantly more common among females (*χ* ^2^ = 7.403, *p* = 0.007). There was no significant difference in presence or type between periods. Average values for the four modeled food groupings differ significantly. There is a significant difference in consumption of C_4_/CAM plants by those individuals with modified heads consuming more (26.5 ± 9.9% vs. 23.6 ± 9.4%, Wilcoxon signed‐rank test, *p* < 0.05). In the Middle Period sample, males consume significantly more C_4_/CAM plants (*p* < 0.05) and females more C_3_ plants (*p* < 0.01). Four of those with outlier C_4_ values were analyzed for strontium, yielding values outside the “local” range.

**Conclusions:**

As head shaping is permanently imposed in infancy while dietary patterns are the consequence of ongoing choices and constraints in the social and ecological environment, these markers of identity are not parallel. The numerous points of intersection between these markers and other aspects of identity are highlighted.

The human body, as well as aspects of identity, are regularly and repeatedly shaped by the interactions of each individual with their society and environment. Some of these interactions create rapid and dramatic shifts in the body and social persona, while others build incrementally over the life course. In all cases, however, an individual's identities intersect with their biology. Individuals will have multiple, complex, and overlapping identities, any of which could be embodied in various ways. Here, we consider cranial vault modification, a deliberate practice enacted in childhood that results in a visible and permanent alteration of head shape, and food consumption, a more quotidian element of identity. Ultimately, both practices embody some form of social identity whose presence and interaction are broadly visible in prehistoric societies.

Head shaping operates within cultural constraints but is widely seen in the prehistoric world. With the practice of head shaping, there are aspects of collective identity imbued not only in the visible signifier (the reshaped head), but also in the customs used to create specific head shapes during infancy or, indeed, in the decision to abstain from modifying the head (e.g., Duncan & Vail, 2018; Torres‐Rouff, 2020). Similarly, social identities are enmeshed with food preparation and consumption practices and in food choices themselves (e.g., Curet & Pestle, 2010; Hastorf, 2017). These presentations of identity, both immutable in the form of head shape, and mutable in the scope of a lifetime of diet, then reflect different and intersecting aspects of any individual's social identities (Torres‐Rouff & Knudson, 2017). The lens provided by body alterations and the patterns manifest in diet make clear that embodiment is an iterative and transformative process that occurs throughout the life course and can be studied in human populations globally (e.g., Ingold & Pálsson, 2013; Joyce, 2005; Meskell, 2001). Therefore, if cranial vault modification serves to signal aspects of shared social identity to the individual and the group, and food in some sense creates and reinforces identity, can we use data from each to discuss the ways that head shape and diet speak to each other and to identity construction more broadly?

Through analysis of head shaping in 1190 crania from the San Pedro de Atacama oases of northern Chile, we document patterns of cranial vault modification in terms of presence/absence, the distribution of different types of modification, and the degree of modification. We briefly explore changes in head shaping practices through time, across cemeteries, and its relationship with biological sex. Once these parameters of early embodiment and identity are defined, we consider their intersection with longer term/cumulative processes of identity formation as viewed through food practices. Specifically, cranial modification data are considered in relation to the results of stable isotope analysis of carbon and nitrogen from 203 individuals from the oases. We use these integrated data sets to explore multiplicity in identities and the potential intersections between the manifestation of crucial child rearing practices in head shaping and the everyday actions of preparing, eating, and sharing meals.

Variations in burials from the San Pedro de Atacama oases provide strong evidence that individuals manifested different identities, practices, and access to resources. Beyond the material record, which shows differences in material wealth as well as in specific items including foreign and ritual goods included in the grave (e.g., Horta, 2014; Llagostera 2004; Núñez, 1992; Plaza & Martinón‐Torres, 2021; Salazar et al., 2014; Torres‐Rouff, 2008; Torres‐Rouff & Knudson, 2017), the individuals themselves embody differences among the population. This manifests, for example, in the ways that some individuals were subject to violent injury in highly visible and scarring ways (i.e., Torres‐Rouff, 2011; Torres‐Rouff et al., 2018) and equally in what seems to have been multiple social distinctions between males and females in dress, diet, and head shape (i.e., Pestle et al., 2016; Pestle, Hubbe, et al., 2021; Stovel, 2001, 2013; Torres‐Rouff, 2008). While there are status differences between individuals interred in oases cemeteries (e.g., Hubbe et al., 2012; Torres‐Rouff et al., 2015), there are no clear categories that make that analysis easily accessible here.

Head shaping and diet, therefore, are both embodied practices that through attention and repetition carry social significance. In both cases, this significance goes beyond the individual to reflect culture and group identity or affiliation. As practices that reflect social interactions and commitments, they can speak to us about the experience of living a life in the prehistoric Atacameño oases and potentially serve as a model for these explorations elsewhere.

In the Andes broadly, as well as in the Atacameño oases more specifically, cranial modification is understood to serve as a marker of identity (e.g., Andrushko, 2021; Blom, 2005; Torres‐Rouff, 2020; Velasco, 2018). The documented patterns in the Atacameño oases indicate individual and cemetery‐level variation, as well as changes over the large spans of time the oases have been occupied (Torres‐Rouff, 2007). The permanence and visibility of head shaping suggest that it codes for something in the child rearing period that continues to be relevant and manifest in adulthood. Archeologically, we cannot access motivation or the specifics of meaning that are associated with any particular modification, or lack thereof, by an individual, family or community. We can, however, see that there was an effort to convey meaning and association, and that there were likely different meanings conveyed by the presence or the absence of modification, the varied forms employed, and in the strength/degree of the modification.

In contrast to head shaping, diet can document communal identity but also may reflect the fluidity of identities over the life course. Despite isotope analyses providing a single data point for the individuals considered here, each of these points reflects years of individual and societal decisions. Diet reveals the regular choices made by an individual, family, or other social group about what to eat, and consequently can communicate key aspects of social identity, including class or social status, (Curet & Pestle, 2010; Fischler, 1988; Guyatt et al., 1993; McDade, 2005; Mintz, 1985; Pottier, 1999; Smith, 2006; Ware, 1987). As Hastorf (2017, p. 3) notes, “Societies are made manifest in their food traditions, recipes, and the daily cycles that meals dictate, formed in the sharing of meals and dishes. These actions create society, which in turn becomes the milieu of identity formation.” In the case of the San Pedro oases, our earlier findings show notable dietary variation in smaller samples (Hubbe et al., 2012; Pestle et al., 2016). As such we expect there to have been identity‐driven dietary variability (particularly in the consumption of meat vs. legumes and C_3_ vs. C_4_ plants) that would be in keeping with archeological and ethnohistoric evidence of linkages between status and the consumption of maize beer (*chicha*) and meat (Duke, 2011; Hastorf & Johannessen, 1993; Jennings, 2004). These patterns of variation reflect ongoing choices as well as the many limitations in the social and ecological environments. Together then, diet and head shape may reveal both shared and distinct identities embodied in the people of the Atacameño oases.

Is it possible therefore, that what we see in diet as a mutable form of identity that stands as a proxy for resource access and some forms of collective identity also has ties to head shape and those immutable forms of identity that are imposed on infants? This raises the idea that multiple axes of identity are intersecting and represented in each individual body. As originally framed by Crenshaw (1989, 1991) intersectionality considers “categories not as distinct but as always permeated by other categories, fluid and changing, always in the process of creating and being created by dynamics of power” (Cho et al., 2013, p. 795). Here we can focus on whether head shape and diet echo similarities in identity construction and embodiment between individuals or if they in fact speak to different aspects of identity at play in each body. As such, this understanding of multiple and fluid social identities intersecting in the body can frame our understanding of the lived experience of people in the prehistoric Atacameño oases. Here, we investigate whether head shaping and diet embody different aspects of identity and social differentiation and if this changes over time, with the more cosmopolitan Middle Period (AD 400–1000) potentially reflecting different tendencies than the more insular Late Intermediate Period (AD 1000–1450). Posed differently, does sharing cranial modification, a particular type, or the lack thereof, connect with, or manifest in, dietary distinctions for the members of this particular society?

## 1 SITE BACKGROUND

**FIGURE 1:**
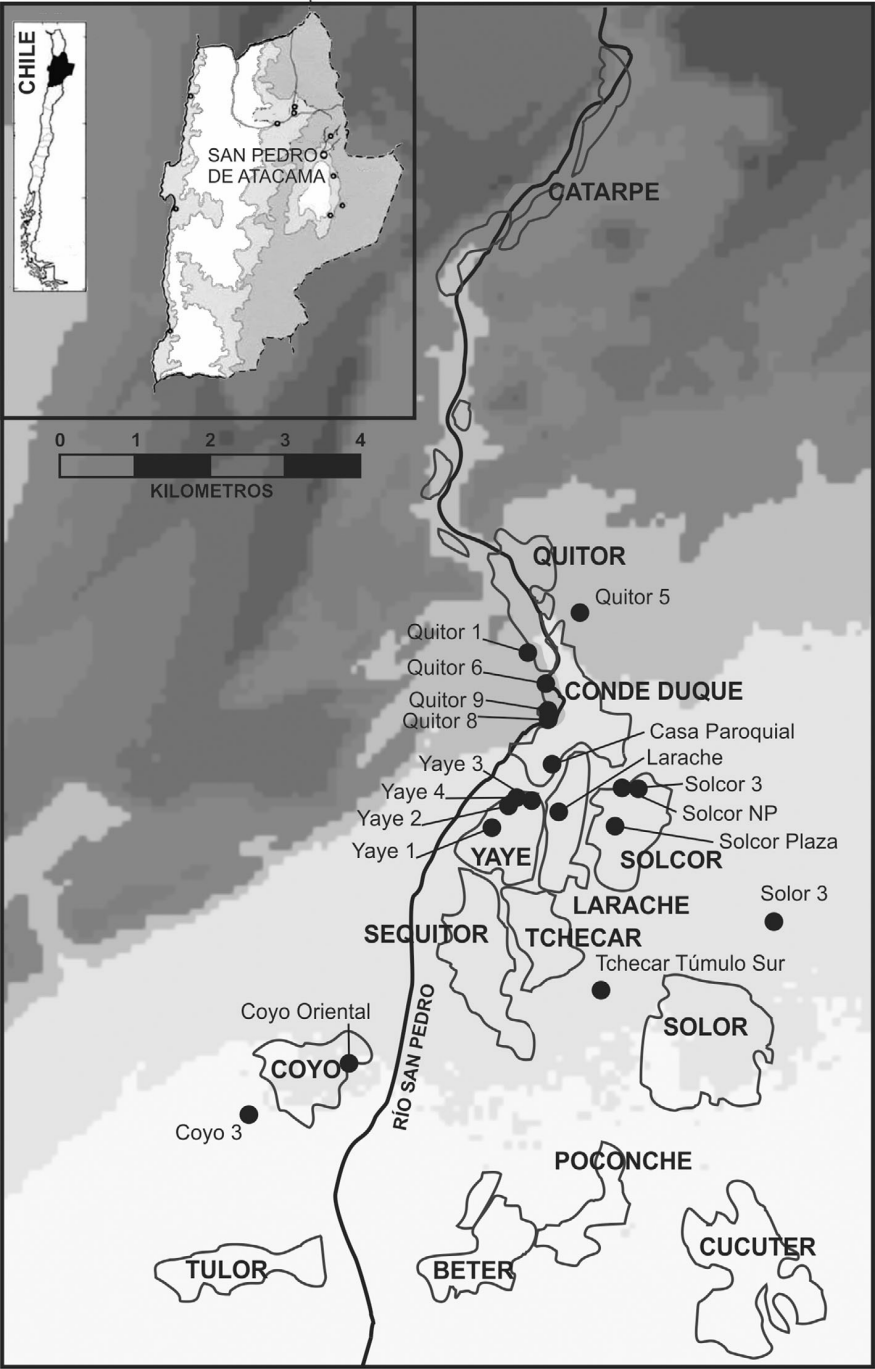
Map of the region with sites indicated in the text

San Pedro de Atacama comprises a series of small oases at 2450 m above sea level in northern Chile's Atacama Desert (Figure 1). During the early occupation of the oases (Formative Period; c. 200 BC) we see the construction of agglutinated villages in place of the earlier pastoralist way of life. There is no strong evidence of social stratification in the early periods (Muñoz, 1989, p. 125). By the Middle Period (AD 400–1000), to which the bulk of our samples date (Pestle, Torres‐Rouff, et al., 2021), the oases included permanent settlements where Atacameños practiced camelid pastoralism and small‐scale agriculture (Llagostera and Costa, 1999; Núñez, 2007). This is bolstered by incipient craft specialization, the growth of agriculture, and individuals who focused their energies on the caravan trade into the interior (Núñez, 1992, p. 55; Pimentel et al., 2007; Salazar et al., 2011). Despite these changes, the Atacama oases are situated in an area where the environment strongly limited food diversity and we see, for example, no evidence for coastal imports figuring large in human diet (Pestle, Hubbe, et al., 2021). Núñez (1992) argued that the Middle Period shows the earliest evidence for surplus production. This phenomenon facilitates the growing caravan trade, a key factor in the rise of persistent inequality. As we have argued elsewhere (e.g., Torres‐Rouff, 2008; Torres‐Rouff et al., 2018), evidence suggests that the abundant prosperity and increasing engagement with local and interregional networks during the Middle Period was not an evenly distributed benefit. During the subsequent Late Intermediate Period (AD 1000–1450), this area witnessed a substantive social reorganization with the collapse of foreign influences from the Bolivian *altiplano*, shifts in cultural practices and material styles, a loss of human biological diversity, consolidation of settlements, and the construction of defensive structures (Llagostera, 2004; Llagostera and Costa, 1999; Mostny, 1949; Torres‐Rouff, Knudson, and Hubbe, 2013).

## 2 MATERIALS

This study incorporates data from a broad sweep of primarily Middle Period (MP; AD 400–1000) as well as some Late Intermediate Period (LIP; AD 1000–1400) skeletal collections in San Pedro de Atacama (Pestle, Torres‐Rouff, et al., 2021; Torres‐Rouff & Hubbe, 2013). As noted earlier, these periods encompass a time of considerable growth and interregional interaction and broad prosperity (MP), followed by the subsequent decline of these (LIP). For this study, 1190 crania from 17 sites were studied for evidence of cranial modification, and 203 individuals from 12 sites were sampled for carbon and nitrogen stable isotope analyses to provide insight into dietary practices (Table 1). Results of the carbon and nitrogen analyses were then deepened with the inclusion of strontium data for a small subset of the sample. The majority of these human skeletal remains were excavated by Father Gustavo Le Paige in the mid‐twentieth century and do not include post‐cranial elements, while those individuals from the sites of Casa Parroquial, Coyo 3, Solcor 3, and Quitor 6 Tardío were excavated following scientific protocols by the staff of the Instituto de Investigaciones Arqueológicas (now Instituto de Arqueología y Antropología) of the Universidad Católica del Norte, Chile in the subsequent decades. All remains are housed at the Museo R.P. Le Paige in San Pedro de Atacama, Chile.

**TABLE 1:** The sample with cranial vault modification details

Site	Time period	Isotope sample	Total skeletal sample	Unmodified	Annular modification	Tabular modification
Casa Parroquial	MP	7	6	3	1	2
Coyo 3	MP	9	43	26	0	17
Coyo Oriental	MP	67	214	123	33	58
Larache	MP	16	47	21	5	21
Quitor 5	MP	15	176	77	29	70
Quitor 6 Tardío	MP	8	39	21	4	14
Quitor 8	MP	2	36	25	5	6
Quitor 9	MP	4	14	9	2	3
Solcor 3	MP	34	119	66	20	33
Solcor NP	LIP	3	6	2	3	1
Solcor Plaza	LIP	21	78	47	22	9
Solor 3	MP	0	63	26	13	24
Tchecar Túmulo Sur	MP	17	198	133	28	37
Yaye 1	LIP	0	42	18	1	23
Yaye 2	LIP	0	62	41	3	18
Yaye 3	LIP	0	24	19	1	4
Yaye 4	LIP	0	23	13	4	6
	MP Sample	179	955	530	140	285
	LIP Sample	24	235	140	34	61
	Total	**203**	**1190**	**670**	**174**	**346**

## 3 METHODS

### 3.1 Cranial vault modification

Crania were analyzed using standard bioarchaeological protocols (e.g., Buikstra & Ubelaker, 1994; Buzon et al., 2005). To provide a demographic profile, sex and age were assessed. Sex was determined based on the sexually dimorphic features of the skull and os coxae in adult remains. Broad age categories (juvenile, 0–18 years; young adult, 18–30 years; middle adult, 30–50 years; old adult, 50+ years) were created based on dental eruption and cranial suture closure for most individuals when post‐cranial remains were unavailable. Where available, data from the os coxae were prioritized for sex and age determinations.

All skulls were analyzed for evidence of the intentional alteration of head shape. Given that cranial modification relies on visible alterations in its role as a social signifier, it likely reflects broad aspects of collective identity and affiliation, and the modification itself provides an immutable marker of this. Modified individuals were grouped into two broad categories: annular (circumferential) or tabular (fronto‐occipital) (e.g., Dembo & Imbelloni, 1938). The circumferential constriction of annular modification produces an elongated, narrow head shape, while tabular modification results from anteroposterior compression and produces a broadened head shape. These types of cranial modification should have been recognizably different in the living. Although substantial variations exist within these classifications, generally related to the angle of pressure, the two primary types reflect structural differences in the practice in terms of the nature of the constriction and the apparatus used to obtain a shape. Minor deviations from the unmodified state were likely undetectable in life and are not considered here. Degree of modification was scored independently for the anterior and posterior of the cranium on a scale from 0 (absent) to 4 (pronounced). This scale is based on a qualitative evaluation of each cranium and the relationship of arc and chord measurements to ascertain flatness and, therefore, degree (Torres‐Rouff, 2008). The most pronounced modifications likely reflect a consistent dedication to the act of modifying a child's head and encompass those individuals with a score of 4 on either the anterior or posterior. For the purposes of this discussion, individuals with only 2 s and 3 s were considered moderately modified while those with a 1/1 or a 1 paired with a 2 were considered as having slight modification, raising the possibility that some modifications could have been obscured by hair or dress in life. The unmodified were scored as 0. As head shaping is a practice, this range helps us to see it as more than a simple typology or a binary distinction between the modified and unmodified. A focus on the presence and distribution of cranial modification allows us to explore the role of head shaping by ascertaining whether time was dedicated to this practice during infancy in such a way that the body was visibly marked for life.

### 3.2 Carbon and nitrogen stable isotope analyses

Stable isotopes of carbon and nitrogen were analyzed to elucidate individual level diet composition. These isotope systems were chosen given their nearly half‐century history of successful archeological application and their ability, when employed using a mixture model, to discriminate among and between the isotopically and compositionally distinct food groups found in the Atacama Desert (e.g., ^13^C enriched C_4_/CAM plants vs. C_3_ plants depleted in ^13^C, or ^15^N enriched animal tissues compared with beans possessing far lower 20^15^N values). While the modeled data from carbon and nitrogen isotopes provide a panorama of dietary choices and not details of change over the lifecourse, these data give a strong sense of individual‐ and group‐level differences and diet. Complete details on stable isotope analysis sampling, extraction, instrumentation, and modeling are presented in Pestle, Hubbe, et al. (2021) and the paleodietary data discussed here are derived without modification from the data presented in that work. Nonetheless, below we provide a brief summary of the methods employed. For the present work, we consider the isotopic data of 203 individuals for whom data on cranial vault modification also was available.

Samples of dense cortical bone were removed from available skeletal elements at the Museo R.P. Le Paige using a diamond cut‐off wheel on a rotary tool. The extraction of target biomolecules (collagen and hydroxyapatite) from samples was performed at the Archeological Stable Isotope Laboratory at the University of Miami. Collagen extraction followed the protocol of Longin (1971), as modified by Pestle (2010), while hydroxyapatite extraction followed a protocol first established in Lee‐Thorp et al. (1989) and Krueger (1991) and modified by Pestle (2010). Isotopic analysis was performed in the Marine Geology and Geophysics Stable Isotope Laboratory at the Rosenstiel School of Marine and Atmospheric Science, University of Miami. Only well‐preserved samples (collagen yield >0.5 wt%, carbon yield >4.5 wt%, nitrogen yield >0.9 wt%, atomic C/N ratio between 2.9 and 3.6) were included in subsequent dietary modeling. Hydroxyapatite integrity was assessed by reference to expected chemical yield range provided in Chesson et al. (2021), although as these samples hail from the driest desert in the world, postmortem alteration via typical pathways (i.e., dissolution and recrystallization) was rare.

The Bayesian model FRUITS (Food Reconstruction Using Isotopic Transferred Signals; [Fernandes et al., 2014]) was used to quantify individual dietary composition. Consumer data for the model were based on the isotope ratios generated by IRMS. To account for fractionation, we first determined the consumer‐foodstuff offset (and error) for δ^13^C_co_ using the method detailed by Pestle et al. (2015). Fractionation of δ^13^C_ap_ was stipulated as 10.1 ± 0.4‰, following Fernandes et al. (2012). Finally, for δ^15^N_co_ we employed a trophic fractionation value of 3.6 ± 1.2‰, as recommended by several experimental studies of omnivorous animals (Ambrose, 2002; DeNiro & Epstein, 1981; Hare et al., 1991; Howland et al., 2003; Sponheimer et al., 2003; Warinner & Tuross, 2011).

Foodweb isotope values comprised the edible portions of 62 plant and animal samples (Table 2). Any modern data included in this reference sample had δ^13^C values corrected by +1.5‰ to account for recent fossil fuel burning (Keeling et al., 1979). Furthermore, the δ^13^C value of bone samples were adjusted by −2.0‰ to account for bone collagen‐edible tissue offset (Krueger & Sullivan, 1984; Lee‐Thorp et al., 1989). While elevated δ^15^N values are a noted feature of arid environments (Ambrose, 1991), as all foodweb samples used in the model were locally derived, the isotopic “grade shift” of the region has no bearing on the functioning of the model. Macronutrient (protein, carbohydrate, lipid) composition of each food group was determined by reference to the USDA Nutrient Data Laboratory (2016). Elemental composition (particularly %C) of each foodstuff/macronutrient group was based on formulae provided in Morrison et al. (2000), and differences in digestibility were accounted for following Hopkins (1981). For modeling, available foodstuffs were divided into four groups reflecting taxonomy and photosynthetic pathway (beans, C_3_ plants, C_4_/CAM plants, and terrestrial fauna).

**TABLE 2:** Foodweb values

	Macronutrient concentration (%)		Tissue δ^13^C (‰)			Tissue δ^15^N (‰)	
Food grouping	Protein	Energy	Bulk	Protein	Energy	Bulk	Protein
Beans	28 ± 6	72 ± 8	−23.5 ± 1.7	−24.2 ± 1.7	−23.3 ± 1.7	0.7 ± 3.0	0.7 ± 3.0
C_3_ plants	11 ± 7	89 ± 11	−23.7 ± 2.0	−22.3 ± 2.0	−23.8 ± 2.0	8.6 ± 5.4	8.6 ± 5.4
C_4_/CAM plants	11 ± 7	89 ± 11	−11.2 ± 1.6	−9.8 ± 1.6	−11.3 ± 1.6	12 ± 5.6	12 ± 5.6
Terrestrial animals	77 ± 13	23 ± 13	−16.7 ± 3.1	−15.0 ± 3.1	−22.5 ± 3.1	9.5 ± 1.8	9.5 ± 1.8

To account for differential routing, all nitrogen in bone collagen was stipulated as coming from dietary protein, the carbon in hydroxyapatite was stipulated as reflecting all dietary car‐ bon, and the carbon composition of bone collagen was set as reflecting a 3:1 ratio of dietary protein to energy (Fernandes et al., 2012). Carbon isotope offsets between measured bulk food isotope values and the isotopic values of a foodstuff's fats (bulk δ^13^C‐6‰) and carbohydrates (bulk δ^13^C + 0.5‰) were based on data from Tieszen (1981). The carbon isotope signature of a measured bulk foodstuff's protein was determined using a mass‐balance equation, such that a proportional/weighted average of the δ^13^C of protein and energy (fats and carbohydrates) would equal the measured δ^13^C bulk value (corrected for the concentration of carbon in each macronutrient and foodstuff‐appropriate macronutrient concentration). Consumption of protein was limited to between 10 and 50% of protein as energy (using the FRUITS a priori data option), reflecting the lower and upper limit of possible human protein intake (Joint WHO/FAO/UNU Expert Consultation, 2007). All FRUITS simulations were performed using 10,000 iterations, as recommended by the program's developers.

The integration of this data allows us to explore the relationship of head shaping and diet as markers of aspects of social identity and the interplay of these factors in individual bodies and community. A consideration of presence and degree of modification, on the one hand, and dietary information, on the other, with the way these patterns may shift over time can provide insight into different aspects of the individual as well as of the social group.

### 3.3 Strontium isotope analyses

We additionally contextualize the relationship between cranial modification styles and paleodiet with radiogenic strontium isotope data from specific individuals. Strontium isotope (^87^Sr/^86^Sr) data in archeological human remains can be used to infer the geologic region or regions in which that individual lived during enamel or bone formation, since the isotopic composition of ^87^Sr/^86^Sr in the bedrock does not change appreciably as it is incorporated into soil and water, then plants and animals and ultimately humans who live in the region, assuming the strontium is from local geologic sources (see overview in Bentley, 2006). The geological variability in the south‐central Andes makes it suitable for radiogenic strontium isotope analysis, and there is a growing body of comparative data (e.g., Barberena et al., 2021; Dahlstedt et al., 2021; Knudson et al., 2014).

Here, we present eight radiogenic strontium isotope values from four individuals buried in the San Pedro de Atacama cemeteries of Larache and Tchecar (Knudson & Torres‐Rouff, 2014; Torres‐Rouff et al., 2015) with outlier carbon isotope values; given dental health and curation practices, not all individuals with bone suitable for carbon and nitrogen isotope analysis also had sufficient enamel for strontium isotope analysis. For all individuals, two samples were collected from two different teeth that formed at different times in the first years of life.

Enamel samples were collected at the Museo R.P. Le Paige and then prepared and analyzed in the Archeological Chemistry Laboratory at the Arizona State University (ASU). Teeth were photographed, cast, and mechanically cleaned before enamel powder samples were collected with a Dremel drill with carbide burrs. Strontium was separated from the sample matrix using EiChrom SrSpec crown‐ether resin (Horwitz et al., 1992). Samples were analyzed using a Thermo‐Finnigan multi‐collector inductively coupled plasma mass in the ASU Metal, Environmental, and Terrestrial Analytical Laboratory, where external standard SRM‐987 yielded ^87^Sr/^86^Sr = 0.71029 ± 0.00003 (2*σ*, *n* = 10).

To determine a local range of radiogenic strontium isotope values, small mammal samples were collected from the San Pedro de Atacama oases (Knudson & Torres‐Rouff, 2014). Taking the mean ^87^Sr/^86^Sr value of the small mammal samples and adding and subtracting two standard deviations generally gives a large, and therefore conservative, local range (Bentley et al., 2004; Price et al., 2002). Using this definition, we define the “local” range in the San Pedro de Atacama oases as ^87^Sr/^86^Sr = 0.7074–0.7079 (Knudson & Price, 2007; Torres‐Rouff et al., 2015).

## 4 RESULTS

### 4.1 Cranial vault modification

An appraisal of cranial modification (Tables 1 and 3) revealed the presence of modification in 520 individuals (*N* = 1190; 43.7%) across sites and time periods. As such, a majority of the population (56.3%) went unmodified into adulthood. The modified individuals were not monolithic in their use of the practice, with different types and degrees of modification practiced and visible throughout in the oases. Of the 520 modified individuals, 174 evidenced circumferential or annular modification, while 346 were modified in a fronto‐occipital or tabular fashion. There were significant differences between modification patterns among cemeteries; this likely reflects local level nuances as we do not see broad patterns such as the monolithic use of a particular type in any space (e.g., Torres‐Rouff, 2008). Focusing on adults, cranial modification was significantly more common among females (*χ* ^2^ = 7.403, df = 1, and *p* = 0.007; 237/487 or 48.7%) but still frequent among males (175/440 or 39.7%). Most modifications were moderate, although 104 individuals (15.5%) showed evidence of strong modification (defined as possessing the highest score in either the anterior or posterior aspect of the cranium). There was a significant difference in the presence of pronounced modification between the sexes, with the great majority being females (58 females vs. 23 males; *χ* ^2^ = 8.181, df = 1, and *p* = 0.004).

**TABLE 3:** Cranial modification patterns among adult individuals by sex

			Unmodified adults		Annular modification		Tabular modification	
Site	Time period	Total sexed skeletal sample	F	M	F	M	F	M
Casa Parroquial	MP	4	1	0	0	1	1	1
Coyo 3	MP	36	10	12	0	0	10	4
Coyo Oriental	MP	183	53	51	15	15	22	27
Larache	MP	42	8	12	2	3	10	7
Quitor 5	MP	130	31	22	14	10	33	20
Quitor 6 Tardío	MP	25	9	5	1	2	6	2
Quitor 8	MP	32	11	11	3	2	5	0
Quitor 9	MP	14	3	6	1	1	1	2
Solcor 3	MP	107	25	33	10	8	16	15
Solcor NP	LIP	3	0	1	1	0	0	1
Solcor Plaza	LIP	57	15	21	9	3	7	2
Solor 3	MP	42	9	7	4	4	8	10
Tchecar Túmulo Sur	MP	149	41	57	18	6	18	9
Yaye 1	LIP	28	4	7	1	0	8	8
Yaye 2	LIP	37	14	10	1	0	5	7
Yaye 3	LIP	20	8	8	1	0	3	0
Yaye 4	LIP	18	8	2	0	3	3	2
	MP Sample	764	201	216	68	52	130	97
	LIP Sample	163	49	49	13	6	26	20
Total	**927**	**250**	**265**	**81**	**58**	**156**	**117**	

While the LIP sample is considerably smaller (*n* = 235; MP sample *n* = 955), comparison of the two periods produced some interesting results. There is no significant difference between the presence of modification between time periods (*χ* ^2^ = 1.274, df = 1, and *p* = 0.259) regardless of the significant cultural shifts reflected in this time (e.g., Castro et al., 2016). Similarly, the relationship between the broad categories of modification (tabular and annular) seems to hold regardless of period, with tabular forms occurring at roughly double the frequency of annular ones (MP: annular 140, tabular 285; LIP: annular 34, tabular 61) and no significant difference between periods (*χ* ^2^ = 0.012, df = 1, and *p* = 0.914). Finally, the distribution of head shaping between the sexes is also consistent with these broader patterns in adult individuals in terms of both presence and type (Table 3).

These data provide a baseline of distribution and shape variation over time and sex against which to consider dietary information. The patterns we see here speak to permanent identities associated with group, self, and family. These may comport or contrast with the patterns revealed via stable isotope analyses that address the potentially evolving identities in the last decade(s) of an individual's life. Below, we present the results of the stable isotope analyses and consider dietary information in relation to the distribution of cranial vault modification patterns.

### 4.2 Isotope analyses

**FIGURE 2:**
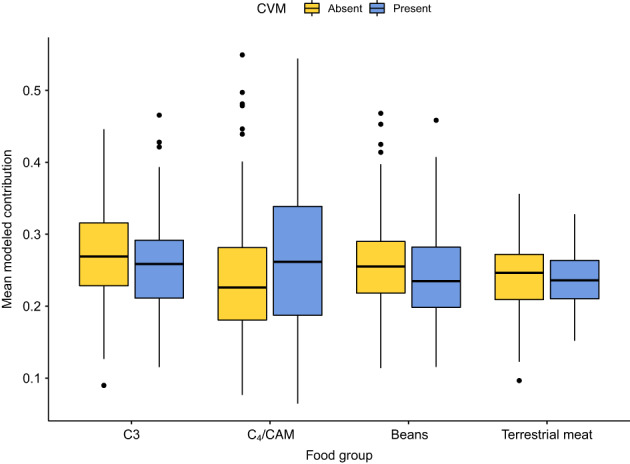
Mean modeled contribution of each food group by the presence or absence of cranial modification

Two hundred and three individuals from both periods ultimately met sample preservation standards and consequently produced reliable estimates of modeled paleodiet. When these data were considered as a whole, they revealed a spectrum of dietary differences among these 203 consumers (Tables 4 and 5). Average values for the four modeled food groupings differ significantly, with mean modeled individual contributions for beans ranging from 11.4% to 46.8%, for C_3_ plants 9.0%–46.6%, for C_4_/CAM plants 6.5%–54.9%, and for terrestrial meat 9.7–35.6%. Comparing dietary data directly to the presence or the absence of cranial modification for all individuals, the only significant difference between modified and unmodified individuals in dietary choice/composition is the higher consumption of C_4_/CAM plants by those individuals with evidence of cranial modification (Figure 2; 26.5 ± 9.9% vs. 23.6 ± 9.4%, Wilcoxon signed‐rank test, p < 0.05). Differences between modified and unmodified individuals do not rise to the level of statistical significance for the other three modeled food groups. A group of seven individuals from the MP (Table 6) was found to have modeled C_4_ contributions that were more than two standard deviations above the mean. Of these, four had enamel that was analyzed for radiogenic strontium. All four yielded values outside the San Pedro de Atacama “local” range of ^87^Sr/^86^Sr = 0.7074–0.7079 (Knudson & Torres‐Rouff, 2014; Torres‐Rouff et al., 2015).

**TABLE 4:** Raw isotope data by site and period

Site	Time period	δ^13^C_co_ (‰)	δ^15^N_co_ (‰)	δ^13^C_ap_ (‰)
Casa Parroquial	MP	−13.3 ± 1.5	12.4 ± 0.8	−7.4 ± 1.0
Coyo 3	MP	−13.0 ± 1.3	11.8 ± 1.2	−8.2 ± 1.3
Coyo Oriental	MP	−14.2 ± 1.2	11.4 ± 0.9	−8.8 ± 1.1
Larache	MP	−13.3 ± 2.3	11.1 ± 1.3	−7.5 ± 2.2
Quitor 5	MP	−15.3 ± 1.9	9.7 ± 1.1	−10.6 ± 2.2
Quitor 6 Tardío	MP	−13.6 ± 1.4	10.8 ± 0.6	−8.1 ± 1.2
Quitor 8	MP	−16.1 ± 0.4	10.6 ± 0.4	−10.6 ± 1.1
Quitor 9	MP	−12.1 ± 1.1	12.0 ± 0.6	−6.9 ± 1.1
Solcor 3	MP	−14.2 ± 1.4	11.4 ± 1.0	−9.0 ± 1.6
Solcor NP	LIP	−14.7 ± 1.2	11.5 ± 0.3	−8.9 ± 1.4
Solcor Plaza	LIP	−13.6 ± 1.3	12.1 ± 0.7	−8.1 ± 1.4
Tchecar Túmulo Sur	MP	−13.9 ± 1.1	11.4 ± 0.5	−8.5 ± 1.0
**MP Sample**		**−14.0 ± 1.5**	**11.3 ± 1.1**	**−8.7 ± 1.6**
*Modified*		*−13.9 ± 1.6*	*11.4 ± 1.2*	*−8.5 ± 1.7*
*Unmodified*		*−14.1 ± 1.5*	*11.2 ± 1.0*	*−8.8 ± 1.6*
**LIP Sample**		**−13.8 ± 1.3**	**12.0 ± 0.7**	**−8.2 ± 1.4**
*Modified*		*−13.4 ± 1.1*	*11.9 ± 0.7*	*−7.8 ± 1.0*
*Unmodified*		*−14.2 ± 1.5*	*12.2 ± 0.7*	*−8.6 ± 1.7*
**Total (all periods)**		**−14.0 ± 1.5**	**11.4 ± 1.1**	**−8.6 ± 1.6**
*Modified*		*−13.8 ± 1.6*	*11.5 ± 1.1*	*−8.4 ± 1.6*
*Unmodified*		*−14.1 ± 1.5*	*11.3 ± 1.0*	*−8.8 ± 1.6*

**TABLE 5:** Modeled contributions by site and period

Site	Time period	C_3_ plants	C_4_/CAM plants	Beans	Terrestrial animals
Casa Parroquial	MP	22.4 ± 3.0	30.2 ± 7.8	20.4 ± 6.3	27.0 ± 2.3
Coyo 3	MP	24.9 ± 5.5	27.6 ± 8.7	22.1 ± 6.4	25.3 ± 3.3
Coyo Oriental	MP	27.1 ± 6.3	24.2 ± 7.6	25.3 ± 4.9	23.3 ± 3.5
Larache	MP	22.0 ± 9.0	31.4 ± 13.7	22.9 ± 9.2	23.8 ± 4.9
Quitor 5	MP	31.0 ± 7.1	15.6 ± 11.4	34.1 ± 9.3	19.3 ± 4.9
Quitor 6 Tardío	MP	23.0 ± 5.9	28.8 ± 10.9	25.0 ± 3.9	23.2 ± 5.0
Quitor 8	MP	40.4 ± 6.0	13.1 ± 2.6	26.1 ± 2.9	20.4 ± 0.5
Quitor 9	MP	21.8 ± 3.8	37.4 ± 9.9	16.2 ± 3.9	24.6 ± 4.5
Solcor 3	MP	27.3 ± 6.4	22.4 ± 8.4	26.0 ± 6.3	24.4 ± 3.9
Solcor NP	LIP	28.3 ± 8.1	22.2 ± 9.1	25.7 ± 6.2	23.8 ± 0.9
Solcor Plaza	LIP	25.5 ± 5.8	27.4 ± 8.4	21.8 ± 5.4	25.4 ± 4.6
Tchecar Túmulo Sur	MP	26.0 ± 4.8	23.6 ± 6.9	24.5 ± 4.7	25.9 ± 4.1
**MP Sample**		**26.4 ± 6.8**	**24.6 ± 9.8**	**25.3 ± 6.9**	**23.7 ± 4.2**
*Modified*		*26.1 ± 7.4*	*26.0 ± 10.2*	*24.5 ± 6.8*	*23.4 ± 3.9*
*Unmodified*		*26.7 ± 6.5*	*23.6 ± 9.5*	*25.9 ± 6.9*	*23.9 ± 4.4*
**LIP Sample**		**25.8 ± 6.0**	**26.7 ± 8.4**	**22.2 ± 5.5**	**25.2 ± 4.3**
*Modified*		*23.9 ± 4.4*	*29.3 ± 7.4*	*21.6 ± 5.4*	*25.2 ± 2.9*
*Unmodified*		*28.1 ± 7.1*	*23.7 ± 8.9*	*23.0 ± 5.8*	*25.2 ± 5.7*
**Total (all periods)**		**26.3 ± 6.7**	**24.8 ± 9.7**	**25.0 ± 6.8**	**23.9 ± 4.2**
*Modified*		*25.8 ± 7.0*	*26.5 ± 9.9*	*24.1 ± 6.7*	*23.7 ± 3.8*
*Unmodified*		*26.8 ± 6.5*	*23.6 ± 9.4*	*25.6 ± 6.8*	*24.0 ± 4.5*

**TABLE 6:** Individuals with outlier C_4_ values

Individual	Sex	Age	Cranial vault modification	^87^Sr/^86^Sr values and dental element analyzed	Modeled C_4_ contribution
Coyo Oriental 5308	Male	Middle adult	Unmodified	–	48.1 ± 11.6
Larache 358	Male	Young adult	Tabular erect	0.71399 (ULC1), 0.71437 (LRM3)	54.4 ± 11.1
Larache 360	Male	Middle adult	Unmodified	0.71413 (ULM1), 0.71427 (ULM2)	54.9 ± 13.5
Larache 390	Male	Young adult	Tabular erect	0.71215 (ULP1), 0.71360 (ULM2)	46.7 ± 16.7
Quitor 5–2179	Male	Old adult	Annular oblique	–	48.7 ± 12.4
Quitor 9–3249	male	Young adult	Unmodified	–	47.9 ± 13.4
Tchecar Túmulo Sur 844	Indeterminate	Middle adult	Unmodified	0.70796 (ULP2), 0.70800 (ULC1)	44.6 ± 13.1

*Note*: San Pedro de Atacama “local” range is ^87^Sr/^86^Sr = 0.7074–0.7079 (Knudson and Price, 2007; Knudson and Torres‐Rouff, 2014). Larache data previously published in Torres‐Rouff et al., 2015; Tchecar data previously published in Knudson & Torres‐Rouff, 2014. Dental elements included in the radiogenic strontium isotope analysis are abbreviated with U = upper or L = lower, then R = right or L = left, then dental element where M = molar, P = premolar, and C = canine.

**FIGURE 3:**
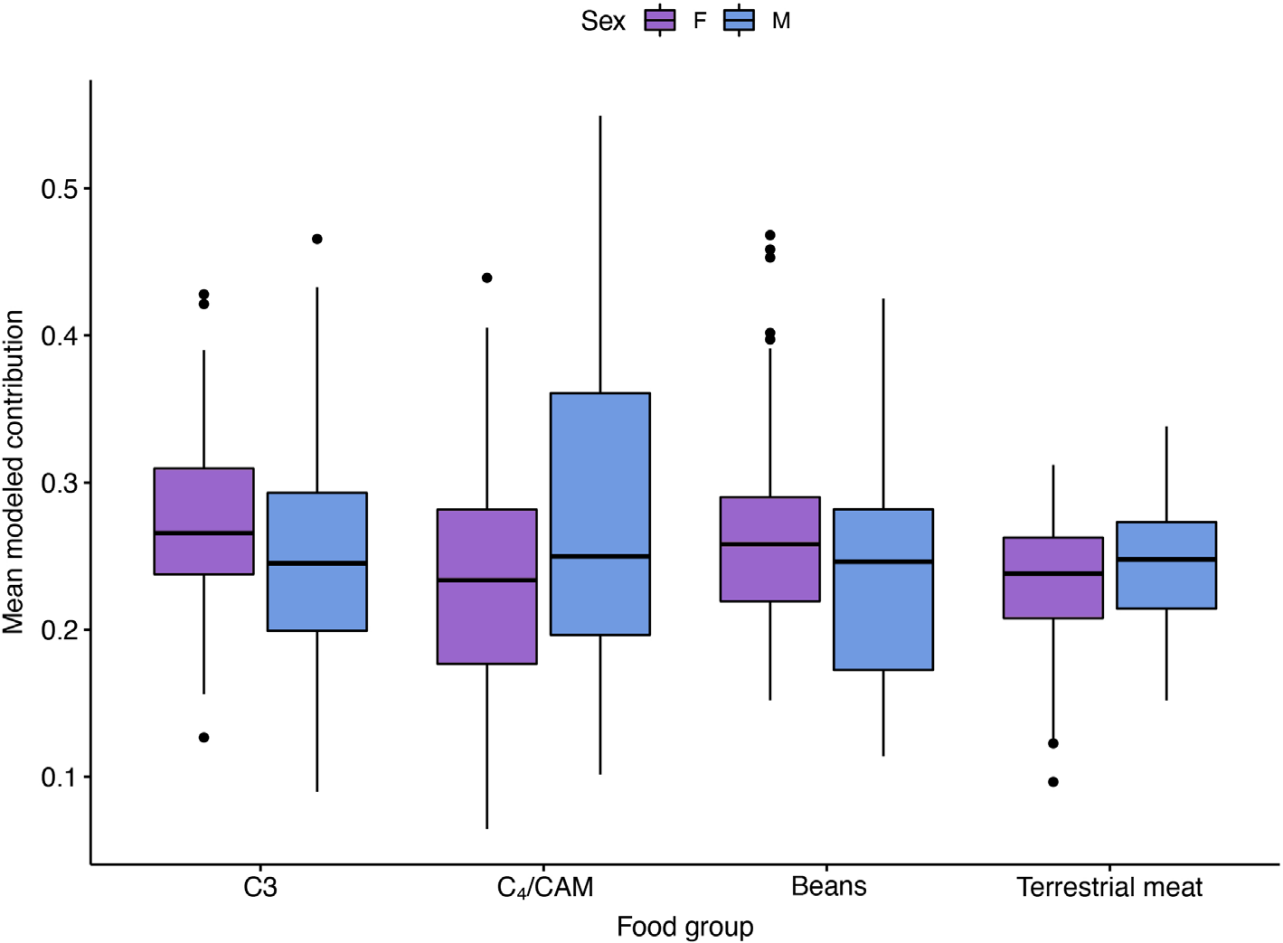
Mean modeled contribution of each food group by sex

**FIGURE 4:**
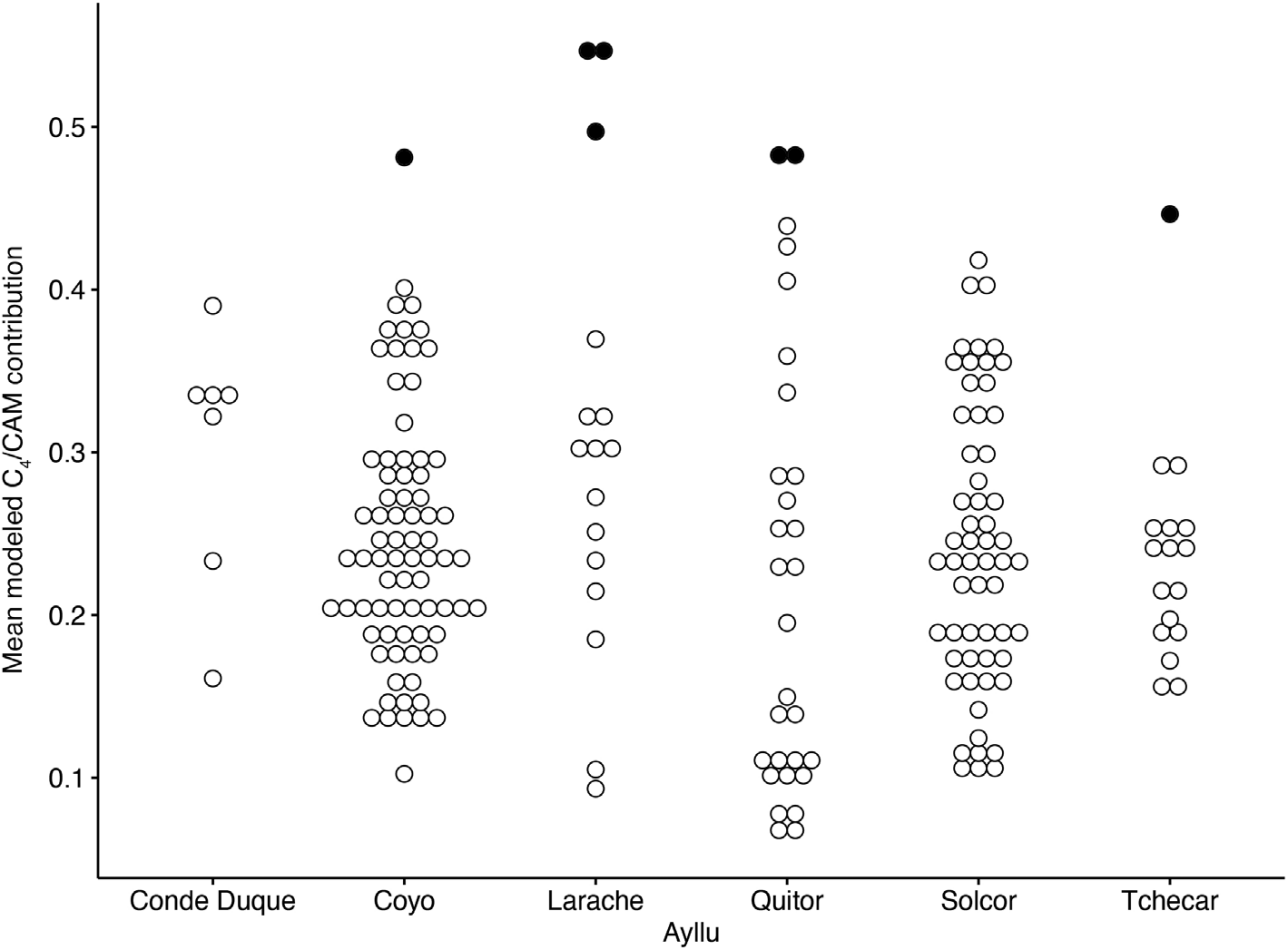
Mean modeled contribution of C_4_/CAM contribution by site location

When sex is added as a factor at this level, unmodified females were found to have consumed significantly more C_3_ plants than unmodified males (Figure 3; Wilcoxon signed‐rank test, *p* < 0.05). There also were significant differences in intra‐group variance in C_3_ consumption among the four sex/modification groups (Levene's test, *p* < 0.05). Similar differences in variance were found among groups for C_4_/CAM plant consumption (Figure 4), and both modified males and females consumed significantly more C_4_/CAM plants than did unmodified females (Wilcoxon signed‐rank test, *p* < 0.05). For legumes, females without modification consumed significantly more (Wilcoxon signed‐rank test, *p* < 0.05) beans than did any of the other three groups (modified females, modified males, unmodified males). This could be an echo of the fact that females in general, regardless of modification, consumed significantly more beans than did males. Interestingly, there were no significant differences in terrestrial meat consumption.

These patterns would appear to reveal two main axes that intersect with diet – sex and head shape. Sex seems to frame a variety of dietary choices in adulthood. For example, males with modified heads consumed a notably higher percentage of C_4_/CAM plants, although the variance in such consumption among males is substantial (from 10.6% to 54.4%), suggesting that multiple factors are at play in maize consumption for men in this sample. While modified females also have a higher rate of C_4_/CAM consumption than unmodified females, they nonetheless consumed less of these foods, on average, than modified males, and showed less internal variance in this consumption.

Taking a more detailed look at those individuals interred during the MP (*n* = 179) we see no significant differences (in average consumption or group variance) between the unmodified and the modified individuals in consumption of any of the four diet categories. Similarly, there were no significant differences in diet between individuals with different types of modification (annular or tabular), although individuals with annular modification had very slightly higher rates of C_4_/CAM consumption and concomitantly lower rates of C_3_ consumption than individuals with tabular forms of head shaping. In keeping with this trend of nonsignificant differences, when degree of modification was examined, there were no significant differences between the unmodified, moderately modified (no scores of 4) and those with pronounced modification (a score of 4 on the anterior or posterior) in terms of dietary composition.

Turning to the relationships among sex, modification, and diet in the MP sample, some significant differences do emerge. The data show that females at this time consumed significantly more C_3_ plants (Wilcoxon signed‐rank test, *p* < 0.01) and legumes (Wilcoxon signed‐rank test, *p* < 0.05) than males, while males consumed more C_4_/CAM foods (Wilcoxon signed‐rank test, *p* < 0.05), and females had significantly lower variance for all three of these food categories (Levene's test, *p* < 0.05 for C_3_ plants and legumes, *p* < 0.001 for C_4_/CAM plants). The patterns were broadly similar when modification was added as an additional factor to comparisons. There were no significant differences in diet between individuals with different types of modification in the MP sample. That said, unmodified females consumed significantly more C_3_ plants than unmodified males (Wilcoxon signed‐rank test, *p* < 0.01) and significantly more legumes than modified males (Wilcoxon signed‐rank test, *p* < 0.01). Unmodified females also consumed significantly less C_4_/CAM plants than males, regardless of modification (Wilcoxon signed‐rank test, modified males: *p* < 0.01; unmodified males: *p* < 0.05). Variances also varied significantly for these two food categories (Levene's test, *p* < 0.05 for C_3_, *p* < 0.01 for C_4_/CAM), with females in general, and unmodified females in particular showing lower variance than their male counterparts.

## 5 DISCUSSION

While cranial modification and diet likely reflect different aspects of social identity given the permanent imposition of one in infancy and the other resulting from ongoing lifestyle choices, there is an intriguing degree of interplay between them. These do not seem to act in tandem, but rather seem to reflect different elements of the social identities of these individuals. A shared form of cranial modification, or the lack thereof, does not seem to be linked to dietary distinctions in these groups despite the fact that diet over the course of life could have been constrained by lineage, sex, or other factors that may have dictated or otherwise been reflected in head shaping choices. Instead, our data suggest that while the imposition of head shaping as a permanent marker in infancy signals some elements of social identity that are also reflected in the ongoing lifestyle choices associated with diet, we do not see clear parallels. Perhaps, this results from other intersecting aspects of identity that gain prominence over the lifecourse or the variable nature of cranial modification that may result from its importance as a child‐rearing practice more than as an adult signifier. It is also important to bear in mind the dietary limitations inherent in the Atacameño oases high desert environment. Indeed, with the limited “menu” of options present, dietary choice may have manifested in very subtle differences in bulk consumption. It may be that food preparation, rather than composition, was a significant identifier that we cannot access through our data. Regardless, the child rearing habits reflected in head shaping do not seem to have extended into social differences marked by obvious dietary choices later in life. Moreover, it is clear that these indicators, diet in particular, tie into sex differences in the Atacameño oases, ultimately signaling a triumvirate of markers (sex, diet, and cranial modification) that shaped and reflected individual lived experiences. We are, of course, unable to access the complete multiplicity of axes of identity intersecting in each individual body, but these lenses allow us to see them at play. Here, we explore these data along two axes: a broader exploration of the relationship that head shape and diet have with sex and then a more detailed view into the notable C_4_/CAM consumption distinctions seen in our sample. Together, these aspects can provide a deeper understanding of the intersection between the body and social persona in the San Pedro oases.

### 5.1 Sex, head shape, and diet

The data presented above reflect a complex relationship between sex and cranial modification and dietary practices. In terms of head shaping, as we have argued in earlier work (e.g., Torres‐Rouff, 2007, 2008), our data demonstrate that neither the presence nor type of head shaping are directly tied to sex in some dichotomous fashion. This coincides with data from Peru where Tung and colleagues (Tung et al., 2016; Tung & Knudson, 2018; Velasco & Tung, 2021) argue that “gender roles and gender norms may not be overtly perceived and performed until later childhood or even puberty” (Velasco & Tung, 2021, p. 2). At some level, this also parallels ethnohistoric documents about the Inka; Guaman Poma, for example, notes gendering most consistently between 9 and 12 years of age, with some practices beginning in the 5‐ to 9‐year‐old category (Lozada & Rakita, 2013, p. 116). It seems that infancy may not have been a time where gender had a strong bearing on a child's lived experience during the Inka period. We would similarly argue that this aspect of child rearing was likely not regimented by a child's perceived gender in the earlier time periods considered here.

That said, it is clear that sex has some kind of relationship to modification practices. The visible distinctions in the increased presence and degree of modification among females in our sample suggest that while the practice does not reflect binary relationships with sex where, for example, individuals in one group are modified while those in another are not, there may be some nuances tied to the way head shaping was practiced on female infants that resulted in these discrepancies. Earlier work in the Atacama has suggested the potential impact of exogamy on cranial modification practices (Costa & Llagostera, 1994), a pattern that we may see at a larger scale here. However, long‐term studies of radiogenic strontium here do not show significant movement of females, calling into question this possibility (Torres‐Rouff & Knudson, 2017). Nevertheless, in the data presented here we do see slight differences in the higher presence of modification among females and the significant difference in degree of modification (Table 7) that would perhaps argue for a specific type and intensity of attention given to female infants over the long process of head shaping by practitioners.

**TABLE 7:** Distribution of degree of modification by sex

	Anterior			Posterior		
Score	Total	Female	Male	Total	Female	Male
**0**	670 (56.3%)	250	265	670 (56.3%)	250	265
**1**	160 (13.4%)	61	61	129 (10.8%)	50	57
**2**	181 (15.2%)	81	62	170 (14.3%)	68	65
**3**	110 (9.2%)	53	37	143 (12.0%)	74	39
**4**	69 (5.8%)	42	15	78 (6.6%)	45	14

Focusing on head shaping as a child rearing practice, we could perhaps consider Tiesler's (2010, p. 191; 2014, pp. 4, 23) argument based on ethnohistoric evidence for the Maya that the practitioners are likely female family members and, as such, note that there may be an aspect visible here where if women bound the heads of infants who are gendered as girls in a particular fashion, that might result in the increased presence of, and perhaps even dedication to, the practice. This, in turn, would produce a more visible alteration in adulthood. Practitioners would make myriad decisions about head shape including techniques to best achieve their goals, local practice, familial preferences, length of use, and so on. These choices, while not made by the child being modified, likely reflect both personal and societal levels of concern and might vary somewhat based on factors that could include gender. The distinctions we see in our sample may reflect some foundational distinctions between individual children that may later resolve into more defined gender identities later in life. Importantly, it is likely that head shape serves to signify other aspects of identity beyond a binary understanding of sex, and that the differences we see reflect a continuum, wherein numerous identities, of both the child and their community, are intersecting.

In contrast to the head shaping data, sex seems to be a prominent aspect shaping food consumption over the course of life. This has previously been implied via dental health studies (Hubbe et al., 2012) showing both sex and status distinctions in caries and dental wear. Here we see a series of notable (but not always significant) distinctions in this sample that primarily revolve around the high rates of legume and C_3_ consumption by women and the elevated consumption of C_4_/CAM plants by men. These sex differences in dietary consumption in the MP remain when head shape is not considered and suggest the overarching role of sex in access to dietary resources. Hastorf (1991, p. 133), for example, argues that gender is the result of “division of labor, differential access, social negotiation, production, and reproduction.” The consumption we see in the oases may in fact reflect this broader construction of gender identity, as potentially gendered access to certain foodstuffs may have been predicated on the complex interaction of patterns of labor, social interactions, and other factors that are difficult to access including status differentials. While C_3_ plants are a dietary staple for populations throughout the region, the significant sex difference in consumption of these and legumes may, at some level, reflect the patterns of large‐scale movement that characterized the MP, particularly if that movement was centered on male driven caravans. This may have resulted in a situation where the females' access to foodstuffs was primarily focused on that which was endemic to the oases, such as beans and C_3_ plants (i.e., algarrobo). Interestingly this does not seem to extend to terrestrial meats, where sex does not seem to have affected consumption practices. Despite the expectation that males traveled with llama caravans as part of the major traffic and exchange networks of the MP in the Atacama (e.g., Clarkson & Santoro, 2021), this suggests that the consumption of terrestrial meats between males and females occurred at the same rates regardless of landscape. This may also put into play the possibility that meat procurement was not a strictly male activity or may have integrated with such things as female herd management.

### 5.2 C_4_ /CAM plant consumption

Finally, our data show interesting patterns related to head shaping and diet when it comes to C_4_/CAM plant consumption in the oases (Table 6). C_4_/CAM plants are consumed more by modified individuals regardless of time period (mean of 26.5 ± 9.9% for the modified vs. 23.6 ± 9.4% for the unmodified). While the distribution of consumption between these two groups does have some overlap, the distinction hints at some kind of dietary restrictions, be they proscriptive or functional. When we focus on the larger MP sample, men consume significantly more C_4_/CAM plants (p < 0.05) while women eat more C_3_ plants (*p* < 0.01). This variability in C_4_/CAM plant consumption suggests that it is neither solely sex nor head shape that is dictating those patterns, but rather that a different axis of variation is at play here, perhaps something tied to status or interregional interaction that can be explored in the future.

Of the seven individuals with particularly elevated C_4_/CAM values (modeled C_4_/CAM contribution Z‐scores >2), three are from the site of Larache, while the other four are distributed across four other cemeteries – Coyo Oriental, Quitor 5, Quitor 9, and Tchecar Túmulo Sur (Table 6). All five cemeteries date to the MP. While there is consistency in that six of these seven individuals are male (one was of indeterminate sex), there are also substantial differences in their bodies and mortuary contexts. The heads of three have been modified, while the other four retain their natural shape. Interestingly, all the modifications, both tabular and annular, are slight. There is no indication of cranial trauma among these individuals, although two graves included potential weapons in the form of a bow and arrow (Quitor 5‐2179) and several axes (a tin ax, a copper ax, and a gold ax with metal bands, potentially of zinc, tin, or silver, all interred with Larache 358; Cifuentes, 2020:284).

Among these individuals, some graves have tremendous material wealth, while others have very few accompaniments in the grave. Larache 358 has some of the greatest material wealth in the oases, including numerous luxury goods and gold items (Torres‐Rouff et al., 2015). In a more typical display of affluence, individual 2179 from Quitor 5 is interred with a variety of goods including hats and varied textiles, a snuff kit, and a variety of utilitarian goods including spinning equipment and ceramic vessels. In fact, 2179 is the only one of these seven to be interred with ritual paraphernalia including snuff trays and tubes. In contrast, Larache 390, Coyo Oriental 5308, Quitor 9–3249, and Tchecar 844 are all interred with few to no grave goods. Together, this suggests there was no uniform quality reflected in the mortuary context that ties them to increased C_4_/CAM consumption, including archeologically visible evidence of elite status.

The three gold *keros* interred with Larache 358 hint at the potential role of *chicha*, a fermented maize beer, in these raised C_4_/CAM levels. Maize *chicha* is associated with elevated status and carries ideological significance throughout the Andes, as well as specifically being understood to carry social weight during the MP (Hastorf, 2003; Jennings, 2004; Logan et al., 2012). Williams and Nash (2021) argue for the use of *chicha* drinking and concomitant feasting as a way of constructing an elite identity among the Wari. As Logan et al. (2012, p. 235) also note, “Chicha…can be made from several plants…but it achieves its highest value if made from maize.” The evidence from Larache 358, both isotopic and material, may suggest that this played a role in this individual's life. That said, the unusual wealth of Larache 358 is aberrant in the oases generally, as well as within this small subset of individuals with elevated C_4_ values (Torres‐Rouff et al., 2015).

Importantly, radiogenic strontium isotope data are available for the three individuals from Larache and the individual from Tchecar with elevated C_4_/CAM values, all of whom show origins outside of the San Pedro de Atacama oases, although the difference is incredibly slight for the individual from Tchecar (Table 6; Knudson & Torres‐Rouff, 2014; Torres‐Rouff et al., 2015, p. 597). This finding raises the broader question of whether these elevated C_4_/CAM values could reflect nonlocal origins and the continuation (either in practice or in the form of isotopic lag) of these particular customs, more than distinctions internal to the lived experience in the oases. Together, these data give hints at a few potential causes for the difference between these individuals and the bulk of the population, again arguing for the ways that multiple axes are at play in the construction, maintenance, and embodiment of social identity.

## 6 CONCLUSION

The suite of data presented here demonstrates that cranial vault modification and dietary choices, both markers of social identity, likely reflect different aspects of the group or even the individual life. As head shaping is permanently imposed in infancy while dietary patterns are a consequence of ongoing choices and constraints in the social and ecological environments, it is not surprising that they do not parallel each other. It is interesting however, that there are numerous points of intersection between these markers of aspects of identity that reflect both early embodiment practices and the transformative quality of identity enacted through meals and diet. The diversity of forms of head shaping in the sample along with the large unmodified portion of the population document the varied ways that this child rearing practice was employed. Our data reveal numerous differences in degree as well as patterns that appear to hold consistent over time in the oases in terms of the presence and type of modification. These serve to reinforce the notion that head shaping does not fit into binary categories of social groups, but rather encompasses a variety of styles and potential meanings. The paleodietary data suggest that similarly complex intersections are at play, and that like with cranial modification, these reflect not only broad social distinctions, but also nuances at the individual level.

In terms of the ways in which we see intersections between cranial modification and diet, it is clear that sex plays some role in the distribution of resources among those individuals studied here. This speaks to what Joyce (2000, p. 162) has denoted as the “mosaic quality of gendered status and power.” While our data suggest that sex plays no consistent role in the presence of an altered head shape, it does appear to have some influence in the degree of modification. This may reflect broader patterns of migration or patrilocality or just the general treatment of female infants in this social context. More strongly, sex seems to shape access to food resources at some level in the oases. The details of C_4_/CAM plant consumption may also speak to Hastorf's (2017:545) assertion that “foods are not only linked to economic value but also to their associated ideological importance.” It is possible that C_4_/CAM plant consumption is integrated into ritual activities surrounding *chicha*, as elsewhere in the Andes, and that that role was limited to a select few. As Gagnon and Juengst (2018, p. 206) argue, “Given the social and political importance of *chicha*, gender, age, status, ethnicity, or other social distinctions in consumption were likely important in the construction of networks and the practices of power.” This would sync with Larache 358 who carries the accoutrements of feasting to the grave in a particularly opulent fashion. However, the intriguing patterns of radiogenic strontium isotope values among the Larache C_4_ outliers could suggest that again what we have here is an intersection of possibilities, the value of maize *chicha* in the ritual sphere overlain on dietary patterns that reflect other places and the influence of sex on consumption. In this way, the variations documented here speak to the numerous intersections of identity and practice, both in child rearing and in food consumption, in and on the body.

Ultimately, our data suggest that these patterns of identity are embodied in such a way that head shape serves as a broad and visible marker of social identity and some sort of assigned and immutable group affiliation that likely has value for the group and individual or family within a community. Diet, meanwhile, in many ways serves as a proxy for ongoing social differentiation, marking individuals by their access to certain resources, or their lack of same, with the flexibility to change over the lifecourse along with an individual's social role. In this way, this embodiment of identities in head shape and diet demonstrates the intersection of multiple individual and group identities together with the transformative nature of social identity.

## AUTHOR CONTRIBUTIONS

**Christina M. Torres:** Conceptualization (lead); data curation (equal); formal analysis (equal); funding acquisition (equal); investigation (equal); methodology (equal); project administration (equal); resources (equal); supervision (lead); writing – original draft (lead); writing – review and editing (lead). **Kelly J. Knudson:** Funding acquisition (equal); investigation (supporting); methodology (supporting); writing – original draft (supporting); writing – review and editing (supporting). **William Pestle:** Data curation (equal); formal analysis (equal); funding acquisition (equal); investigation (equal); methodology (equal); project administration (equal); resources (equal); writing – original draft (supporting); writing – review and editing (supporting).

## FUNDING INFORMATION

This work consolidates information generated under NSF (BCS‐0721229, BCS‐1359644, BCS‐1358753) and FONDECYT 1120376.

## CONFLICT OF INTEREST

The authors declare no conflict of interest.

## Data Availability

The data employed in this study are available in earlier publications (Knudson and Torres‐Rouff, 2014; Pestle et al., 2021a, 2021b; Torres‐Rouff, 2008, 2011; Torres‐Rouff and Knudson, 2017; Torres‐Rouff et al., 2015, 2018) or available from the corresponding author upon request.
